# The influence of parental involvement on students’ math performance: a meta-analysis

**DOI:** 10.3389/fpsyg.2024.1463359

**Published:** 2024-12-17

**Authors:** Xueshen Wang, Yun Wei

**Affiliations:** Faculty of Education, Languages, Psychology & Music, SEGi University, Kota Damansara, Malaysia

**Keywords:** parental involvement, students, math performance, influence, meta-analysis

## Abstract

**Introduction:**

Many studies have confirmed that parental involvement can affect students’ academic performance, but few focus on the influence of parental involvement on students’ math performance by using meta-analysis. This meta-analysis investigates the influence of parental involvement on students’ math performance, along with their moderators.

**Methods:**

Through searching Google Scholar, ERIC, EBSCO, Web of Science and ProQuest databases, a total of 25 empirical studies between 2015 to 2024 were published and 42 independent effect sizes were included. The estimation of effect size was obtained by converting the Fisher’s correlation coefficient and investigating the publication bias that affects meta-analysis studies. This study also conducted heterogeneity tests of the magnitudes grouped according to different moderators.

**Results and discussion:**

The results found parental involvement to had a significantly positive influence on the math performance of students. The analysis of moderating variables found participant, involvement type, grade level, geographical region, and evaluation content all had moderating effects. Finally, the research findings were discussed and suggestions were provided for how parents can be more effectively involved in students’ mathematical learning.

## 1 Introduction

Parental involvement is an important factor that affects the academic performance of students and significant attention has been paid to it by many scholars (Otani, 2020; Olivar and Naparan, 2023). Epstein et al. (2018) stated that parental involvement includes various forms, and different forms of involvement are interrelated, aimed at promoting students’ learning and development. In comparison to other subjects, many students face greater difficulties and pressure to learn mathematics (Rameli and Kosnin, 2016; Waswa and Al-kassab, 2022). To help their children achieve better performance in math learning, parents should be encouraged to be more involved in their mathematical learning, including helping them with homework, communicating with schools and teachers, and providing them with learning support and expectations (Rodríguez Martínez et al., 2017; Jay et al., 2018).

Several studies have shown parental involvement to have an influence on the math performance of students, but the conclusions researchers have reached regarding whether this effect is positive or negative are inconsistent (Silinskas and Kikas, 2019; Panaoura, 2021). Some researchers have stated that parental involvement positively influences the math performance of students (Enih, 2018; Moon, 2020; Koepp et al., 2022), while others have argued that it has a negative influence (Viljaranta et al., 2018; Abu Bakar et al., 2021; Park et al., 2023). Fiskerstrand (2022) argued that it is necessary to conduct a meta-analysis on the influence of parental involvement on students’ math performance. Therefore, a meta-analysis on the influence of parental involvement on the math performance of students is conducted in this study with the aim of answering the following questions:

1. What is the influence of parental involvement on students’ math performance?
2. Whether the influence of parental involvement on students’ math performance is moderated by a variety of variables?

### 1.1 Parental involvement and students’ math performance

The effects of parental involvement on students’ math performance is influenced by various factors, such as the involvement type and parental expectations (Huang et al., 2021; Tang and Tran, 2023). Therefore, it is necessary to clarify the factors that affect the effects of parental involvement on students’ math performance, rather than simply focusing on the quantity and frequency of parental involvement.

The Self-Determination Theory (SDT) provided a valuable theoretical framework for understanding how parental involvement influenced students’ math performance. According to SDT, parental involvement can be divided into positive involvement and negative involvement. Positive parental involvement emphasis on cultivating students’ autonomy in learning and can enhance their intrinsic motivation and learning engagement. When parents provide autonomy support for their children’s learning, it will have a positive influence on students’ academic performance and cultivate their self-efficacy (Joussemet et al., 2008; Feng et al., 2019). On the contrary, excessive control brought about by negative parental involvement can undermine students’ learning autonomy, leading to their inability to truly participate and grasp their own learning, thereby having a negative influence on their academic performance (Xu et al., 2018; Silinskas and Kikas, 2019).

In addition, SDT emphasized that students need to feel connected and supported by others. Parental involvement can cultivate strong emotional bonds and enhance students’ sense of belonging, which is crucial for their motivation and engagement in learning (Ryan and Deci, 2000; Klein, 2019). When parents actively participate in their children’s education while maintaining a supportive relationship, it can improve academic performance of students.

### 1.2 Type of parental involvement

There has long been a lack of a unified concept regarding the definition of parental involvement (Fan and Chen, 2001). Grolnick and Slowiaczek (1994) argued that parental involvement refers to the resources parents invest in their children in a specific field. LaRocque et al. (2011) suggested that it refers to the behavior of parents in the family and school environment as they aim to support the progress of their children in learning. Epstein (2004) pointed out that parental involvement includes six key types: parenting, communication, volunteer service, home-based learning, decision-making, and community collaboration. Generally, parental involvement refers to a situation in which parents participate in the education of their children directly.

Some researchers have focused on the influence of general parental involvement on student math performance in previous studies, while others have focused on the influence different types of parental involvement have on it, such as providing support and expectations for their children during the education process, actively participating in school activities, and helping their children with homework (Hill and Tyson, 2009; Jeynes, 2012; Veas et al., 2019). The influence different types of parental involvement have on the math performance of students is not consistent (Froiland and Davison, 2016; Abah et al., 2018; Viljaranta et al., 2018; Curtis et al., 2021; Alam and Dubé, 2023; Park et al., 2023), so this study uses type of parental involvement as a potential moderating variable.

### 1.3 Participant

In terms of the influence of parental involvement on the academic performance of students, the majority of people tend to consider it from the perspective of the mother (Hindman and Morrison, 2012; Powell et al., 2012). In recent years, as the status of women has improved and attitudes have changed, a growing number of fathers have become involved in the education of their children (Clark, 2005). There is currently no consensus regarding whether or not there is a difference between paternal and maternal involvement. Fathers generally spend more time interacting with their children rather than being involved in other guardianship activities such as feeding or taking care of them (Craig, 2006). In comparison to mothers, fathers exhibit less stimulation when interacting with their children (Belsky et al., 1984). When a mother shows below-average support, the support provided by the father may be particularly important for the child (Martin et al., 2010). Therefore, this study identifies participant as a potential moderating variable.

### 1.4 Grade level

Skaliotis (2010) noted that the grade level of a student can regulate the relationship between parental involvement and their academic performance. From the perspective of cognitive psychology, many studies emphasized that parental involvement and family learning environment can significantly affect students’ cognitive development and academic performance (Landry, 2008; Roy and Giraldo-García, 2018). The types of parental involvement change with the age and cognitive development of students. From parent–child interaction in the preschool stage to academic involvement and assistance in primary school, and then to emotional communication during adolescence, the influence of parental involvement on students’ academic performance varies (Tamayo Martinez et al., 2022; Prime et al., 2023). In addition, for mathematics learning, the knowledge learned in preschool and primary school is relatively simple, while the knowledge learned in middle and high school is much more difficult, which also affects the types of parental involvement (Olmez and Ozel, 2012). Therefore, this study explores the moderating effect of grade level on the relationship between parental involvement and the math performance of students.

### 1.5 Geographical region

Geographical region may also influence the relationship between parental involvement and students’ math performance. The cultural differences in different geographical regions affect the frequency and type of parental involvement in students’ mathematical learning (Erdem and Kaya, 2020; Xu et al., 2024). Some researchers stated that in Asian cultures, parents may have higher expectations and more direct involvement in their children’s education compared to Western cultures, which is positively correlated with students’ math performance (Huang et al., 2021; Tang and Tran, 2023). In addition, the level of economic development and education policies in different regions can also affect the relationship between parental involvement and students’ math performance (Harju-Luukkainen et al., 2020; Wang et al., 2024). Therefore, this study identifies geographical region as a potential moderating variable.

### 1.6 Evaluation type of math performance

Wilder (2023) highlighted that academic performance evaluation method can affect the strength of the relationship between parental involvement and academic performance. Different evaluation types for the math performance of students may also yield different results. If there are problems with academic achievement evaluation, the credibility of any conclusions will decrease (Andrews and Harlen, 2006). Academic performance evaluation is mainly divided into standardized test and custom test. Standardized test generally has a stricter confidence interval and a smaller test score standard deviation, while custom test is easily influenced by many factors or biases of the evaluator. When using custom test to report student math performance, a significant correlation may be found between parental involvement and student math performance (Jeynes, 2005). Therefore, this study explores whether math performance evaluation type has an influence on the relationship between parental involvement and math performance.

### 1.7 Evaluation content of math performance

Math knowledge is not a single component and it encompasses many aspects, including understanding concepts, calculation skills, problem-solving, and algebraic reasoning (Milburn et al., 2019). Conceptual understanding and calculation skills are fundamental knowledge that requires lower personal abilities from students, which makes it easier for students to learn. Problem-solving and algebraic reasoning belong to advanced knowledge and require high personal abilities from students, so some may experience difficulties in learning. Currently, there is a lack of consensus on whether parental involvement has the same influence on different types of math knowledge (Purpura et al., 2020). Therefore, considering whether evaluation content can affect the relationship between parental involvement and math performance is necessary.

### 1.8 Publication year

Study publication year may moderate the relationship between parental involvement and math performance. The rapid development of information technology in modern society has brought more content and forms to the mathematics learning of students, particularly the development of related learning software and the popularization of online learning, which have enabled students to learn mathematics at home (Sarmiento, 2017). This also provides parents with greater opportunities to be involved in the learning of their children. Following the COVID-19 pandemic, parental involvement has faced more opportunities and challenges. Many national and local policies have also started advocating the importance of parental involvement in education and encouraged parents to play a greater role in education, which will help improve the academic performance of their children (Panaoura, 2021). As a result, the relationship between parental involvement and student math performance may become stronger.

### 1.9 Purpose of this study

In summary, the aim of this meta-analysis is to quantitatively integrate previous research results, provide an updated and comprehensive perspective on the influence parental involvement has on math performance, and investigate the aspects that will affect this relationship.

Firstly, this study will calculate the overall effect size of the correlation between parental involvement and math performance. It will then examine whether there are differences in this correlation between involvement type, participant, grade level, geographical region, evaluation type, evaluation content, and publication year.

## 2 Methods

### 2.1 Literature search and inclusion

The literature search and inclusion of this study were jointly conducted by two authors. This study used electronic retrieval to collect English journals and dissertations (government documents, conference papers, etc., were not included in the search scope) from 2015 to 2024 regarding the relationship between parental involvement and students’ math performance (the last retrieval date was May 8, 2024). The databases searched included: Google Scholar, ERIC, EBSCO, Web of Science and ProQuest.

Two rounds of literature searches were conducted. The literature search went through two rounds of procedures. The first round involved conducting extensive searches using keywords. During the search process, we found that although there were many related articles on the relationship between parental involvement and students’ academic performance, these articles often used comprehensive scores combined with other subjects (such as GPA) for students’ math performance. In order to conduct a more in-depth study on the influence of parental involvement on students’ math performance, we used the following search formula, combined with three search fields: topic, title, and full text: (parental involvement OR parental engagement OR parental participation) AND (math performance OR mathematics performance OR math achievement OR mathematics achievement OR math outcomes OR mathematics outcomes).

In the first round, a large-scale search was conducted, and 957 articles were obtained. In the second round, retrospective literature search was used, and 5 articles were obtained by searching the references of the literature obtained in the first round. Following the deletion of 323 duplicate articles, 639 articles were obtained.

**Figure 1:**
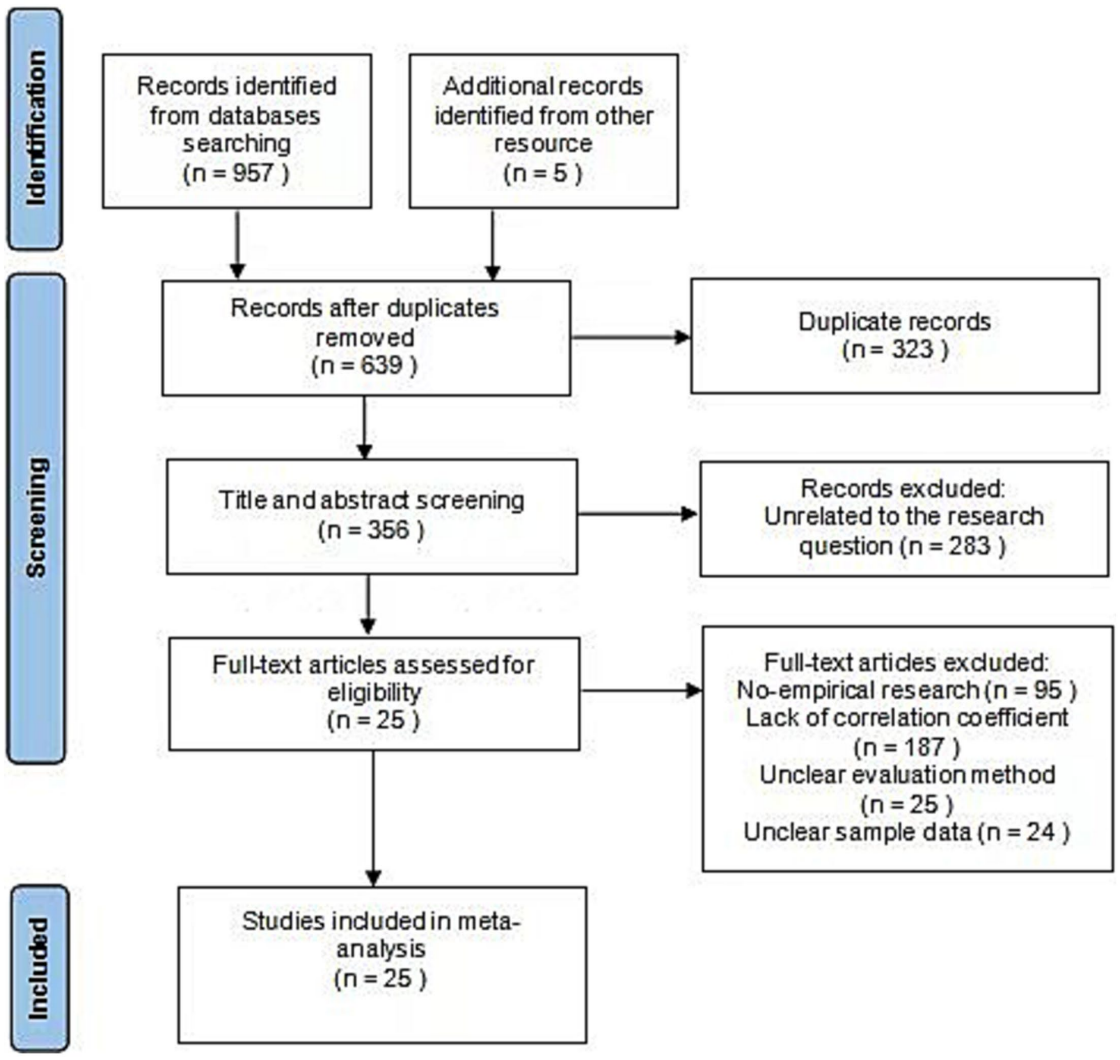
Literature screening process.

In the first round of screening, 283 pieces of irrelevant literature were excluded by reading the article title and abstract. The following criteria were used for screening articles in the second round: (1) articles had to be empirical studies, review studies were not adopted; (2) sample size was clearly reported; (3) the evaluation methods and content for math performance were reported; and (4) the correlation coefficient between parental involvement and the math performance of students was clearly reported. 25 articles with a total of 42 effect size were ultimately included in this study (see Figure 1).

### 2.2 Literature coding

For the convenience of data analysis, the included articles in the meta-analysis needed to be encoded. The coding content included author, effect size, sample size, participant, involvement type, grade level, geographical region, math performance evaluation type, evaluation content, and publication year.

1. Author: The first author was used to code the author of the article, and if an article contained multiple effect sizes, ①, ②, ③ were used to distinguish them.
2. Participant: Father, mother, and all
3. Involvement type: Some articles stated the types of parental involvement, including involvement at home, involvement at school, parental support, parental expectation, and homework assistance. Articles that did not indicate the type of involvement were coded as general involvement.
4. Grade level: Preschool, primary school, middle school, high school, and university.
5. Geographical region: Asia, Europe, United States, and other
6. Math performance evaluation type: According to the description of evaluation methods in the article, this was divided into standardized test and custom test.
7. Evaluation content: Some articles stated the types of math knowledge, including concepts and numbers, calculations, problem-solving, algebraic reasoning, spatial sense, and number sense. Articles that did not indicate the type of math knowledge were coded as general knowledge.
8. Publication year: 2015–2024

To ensure coding accuracy and reliability, the coding in this study was completed by two authors separately. Cohen’s kappa coefficient was used to analyze the consistency of the two authors coding results. According to statistics, the coding consistency rate between two authors was 95.1%. Then, the two authors discussed their differences, reached a consensus, and completed the final codes. The final coding results can be seen in Tables 1, 2.

**Table 1:** Summary of studies included in the meta-analysis (1).

Author	Effect size	Sample size	Father or mother	Involvement type	Grade level
Abah et al.	0.17	73	All	Involvement in home	Primary school
Abodunrin	0.085	454	All	Parental support	Middle school
Akkus & Lynch ①	0.17	41	All	General involvement	Primary school
Akkus & Lynch ②	−0.16	49	All	General involvement	Middle school
Alam & Dubé ①	0.434	117	All	Parental expectation	Primary school
Alam & Dubé ②	0.383	117	All	Parental expectation	Primary school
Alam & Dubé ③	0.312	117	All	Parental expectation	Primary school
Alam & Dubé ④	0.347	117	All	Parental expectation	Primary school
Alam & Dubé ⑤	0.234	117	All	Parental expectation	Primary school
Anicama et al.	−0.03	258	All	Involvement at school	Primary school
Curtis et al.	−0.05	210	All	Involvement at school	Primary school
Foster et al. ①	0.21	581	Mother	Involvement in home	Preschool
Foster et al. ②	0.08	382	Father	Involvement in home	Preschool
Froiland & Davison	0.34	18,623	All	Parental expectation	High school
Grover	0.09	1,468	All	Involvement at school	High school
Koepp et al. ①	0.18	2,167,729	All	General involvement	Primary school
Koepp et al. ②	0.03	1,782,899	All	General involvement	Middle school
Lee & Simpkins	0.12	14,580	All	Parental support	High school
Lee & Rispoli	0.06	1,354	Father	Involvement at school	Preschool
Birgin & Peker	0.431	306	All	Parental support	Middle school
Park et al. ①	−0.21	563	All	Homework assistance	Primary school
Park et al. ②	−0.12	1,613	All	Homework assistance	Middle school
Viljaranta et al.	−0.36	365	Mother	Homework assistance	Primary school
Wu et al.	−0.16	483	All	Homework assistance	Primary school
Silinskas & Kikas	−0.11	512	Mother	Homework assistance	Primary school
Rodríguez Martínez et al. ①	0.287	897	All	Parental expectation	Primary school
Rodríguez Martínez et al. ②	−0.076	897	All	Homework assistance	Primary school
Alghazo & Alghazo ①	0.228	151	All	Involvement at school	Primary school
Alghazo & Alghazo ②	0.247	151	All	Involvement at home	Primary school
Wang, H. et al.	0.06	253	All	Involvement at home	Primary school
Nora’asikin Abu Bakar et al.	−0.146	284	All	General involvement	University
Moon ①	0.2	1,321	All	General involvement	Primary school
Moon ②	0.2	2,916	All	General involvement	Primary school
Moon ③	0.36	548	All	General involvement	Primary school
Moon ④	0.19	1,323	All	General involvement	Middle school
Moon ⑤	0.22	3,036	All	General involvement	Middle school
Moon ⑥	0.33	461	All	General involvement	Middle school
Enih ①	0.22	200	All	Involvement at home	Middle school
Enih ②	0.11	200	All	Involvement at school	Middle school
Sudit ①	0.17	8,565	All	Involvement at school	Primary school
Sudit ②	−0.1	8,565	All	Involvement at home	Primary school
Núñez et al.	0.01	1,250	All	Homework assistance	Middle school

**Table 2:** Summary of studies included in the meta-analysis (2).

Author	Geographical region	Evaluation type	Evaluation content	Publication year
Abah et al.	Other	Custom test	General knowledge	2018
Abodunrin	USA	Standardized test	General knowledge	2021
Akkus & Lynch ①	Asia	Standardized test	General knowledge	2022
Akkus & Lynch ②	Asia	Standardized test	General knowledge	2022
Alam & Dubé ①	Other	Custom test	Calculations	2023
Alam & Dubé ②	Other	Custom test	Problem-solving	2023
Alam & Dubé ③	Other	Custom test	Concepts and number	2023
Alam & Dubé ④	Other	Custom test	Algebraic reasoning	2023
Alam & Dubé ⑤	Other	Custom test	Spatial sense	2023
Anicama et al.	USA	Standardized test	Calculations	2018
Curtis et al.	USA	Standardized test	Calculations	2021
Foster et al. ①	USA	Standardized test	Concepts and number	2016
Foster et al. ②	USA	Standardized test	Concepts and number	2016
Froiland & Davison	USA	Custom test	Algebraic reasoning	2016
Grover	USA	Standardized test	General knowledge	2016
Koepp et al. ①	Other	Standardized test	General knowledge	2022
Koepp et al. ②	Other	Standardized test	General knowledge	2022
Lee & Simpkins	USA	Standardized test	Algebraic reasoning	2021
Lee & Rispoli	USA	Standardized test	Concepts and number	2019
Birgin & Peker	Asia	Standardized test	Number sense	2024
Park et al. ①	USA	Standardized test	General knowledge	2023
Park et al. ②	USA	Standardized test	General knowledge	2023
Viljaranta et al.	Europe	Standardized test	Calculations	2018
Wu et al.	USA	Standardized test	General knowledge	2022
Silinskas & Kikas	Europe	Standardized test	General knowledge	2019
Rodríguez Martínez et al. ①	Europe	Standardized test	General knowledge	2017
Rodríguez Martínez et al. ②	Europe	Standardized test	General knowledge	2017
Alghazo & Alghazo ①	Asia	Standardized test	General knowledge	2015
Alghazo & Alghazo ②	Asia	Standardized test	General knowledge	2015
Wang, H. et al.	Asia	Standardized test	General knowledge	2023
Nora’asikin Abu Bakar et al.	Asia	Standardized test	General knowledge	2021
Moon ①	USA	Standardized test	General knowledge	2020
Moon ②	USA	Standardized test	General knowledge	2020
Moon ③	USA	Standardized test	General knowledge	2020
Moon ④	USA	Standardized test	General knowledge	2020
Moon ⑤	USA	Standardized test	General knowledge	2020
Moon ⑥	USA	Standardized test	General knowledge	2020
Enih ①	USA	Standardized test	General knowledge	2018
Enih ②	USA	Standardized test	General knowledge	2018
Sudit ①	USA	Custom test	General knowledge	2018
Sudit ②	USA	Custom test	General knowledge	2018
Núñez et al.	Europe	Custom test	General knowledge	2017

### 2.3 Effect size calculation

This study adopted the meta-analysis method, which is a comprehensive statistical analysis that is based on existing research results (Borenstein et al., 2021). Firstly, the initial effect size of each study was extracted, which is the correlation coefficient r between parental involvement and the math performance of students. Fisher’s Z-transform was then applied to r and weighted based on the sample size with 95% confidence.

For intervals, the calculation formula was Z = 0.5 × ln [(1+ r)/(1-r)].

The instrument used in this study was Comprehensive Meta Analysis 3.0 software, which is specifically designed for calculating the effect size and total effect size of each original study, and analyzing the heterogeneity of effect sizes in different contexts based on moderating variables.

## 3 Results

### 3.1 Overall assessment and heterogeneity test

**Figure 2:**
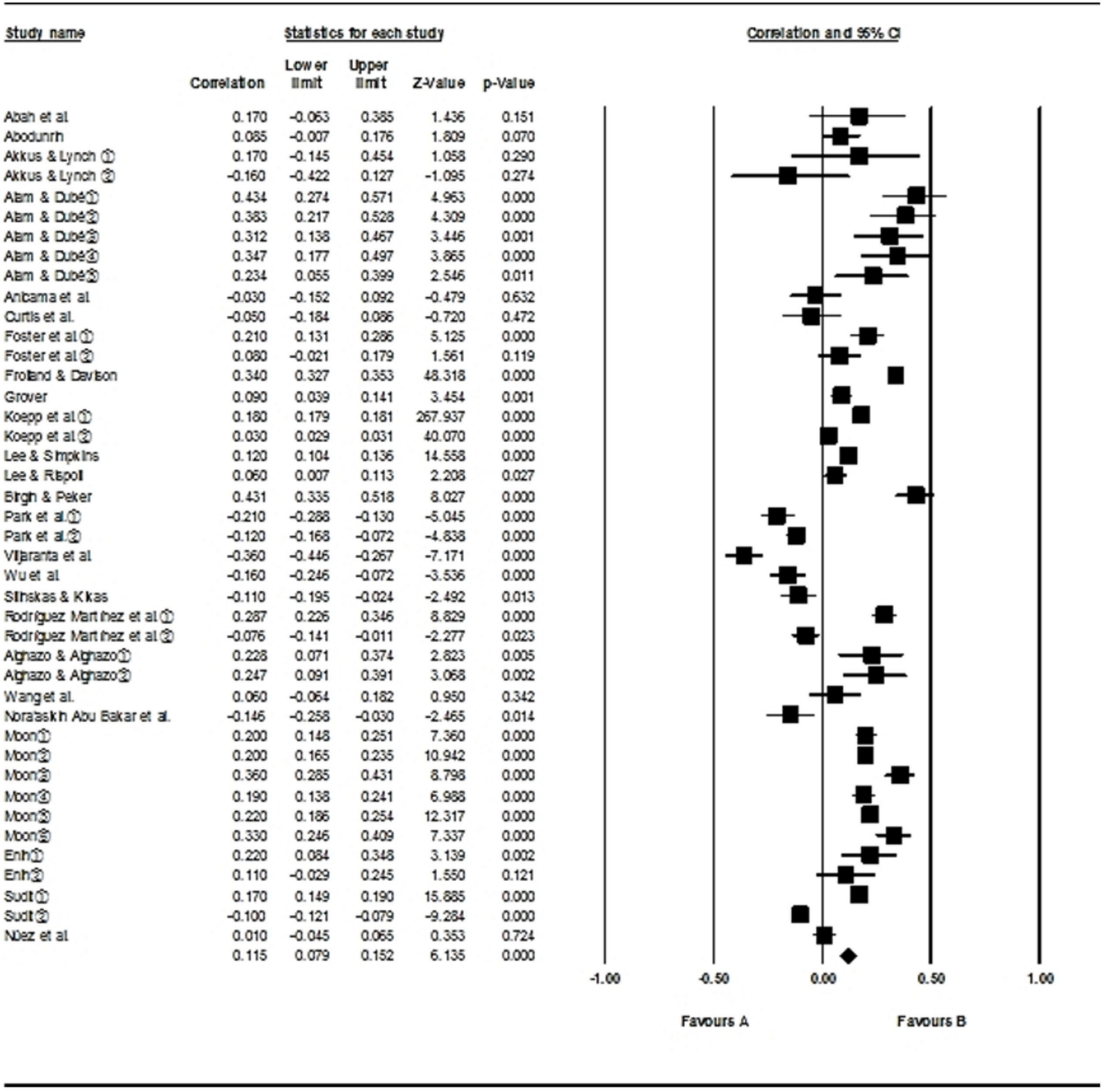
Forest plot.

This meta-analysis included 25 articles with 42 independent effect sizes, with sample sizes ranging from 41 to 2,167,729 for each study. The overall effect size and heterogeneity analysis results of parental involvement on mathematical performance are shown in Table 3 and the forest plot (Figure 2). There are two indicators for a heterogeneity test: *Q* and *I^2^*. When *Q* is significant and *I^2^* > 75%, it is considered that there is significant heterogeneity between studies, and the random-effect model should be chosen for analysis (Huedo-Medina et al., 2006).

**Table 3:** The effect size of parental involvement on math performance.

	*k*	*r*	95% CI for *r*	Homogeneity test			Tau-squared	
				*Q*	*p*	*I^2^*	*Tau^2^*	*Tau*
PI	42	0.115	[0.079, 0.152]	24730.955	0.000	99.834	0.012	0.109

PI, Parental involvement; k is the number of independent effect size.

The results showed a *Q* value of 24,730.955, with *p* < 0.001, and an *I^2^* value of 99.834%, which indicates significant heterogeneity among studies, so the random-effect model was used for analysis. The random-effect model showed the overall effect size of parental involvement on math performance to be 0.115 (*p* < 0.001), with a 95% confidence interval of 0.079 to 0.152. This proved that parental involvement has a significantly positive influence on the math performance of students.

### 3.2 Publication bias assessment

**Figure 3:**
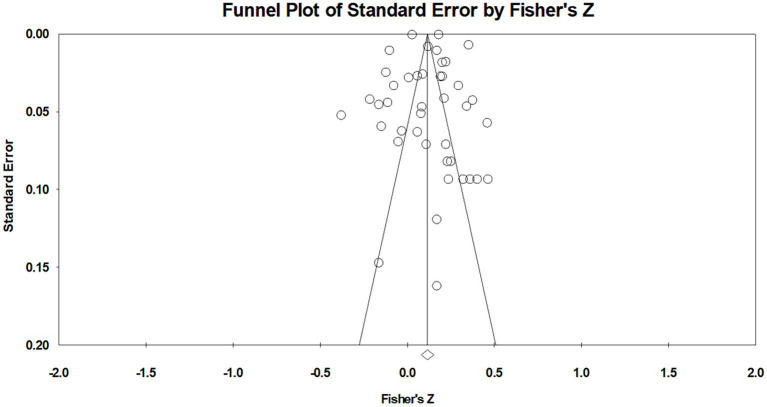
Funnel plot.

Meta-analysis requires a publication bias assessment as a means of ensuring research result reliability. Publication bias assessment in this study involved two steps. Firstly, the funnel plot (Figure 3) showed that the effect sizes of the studies were evenly distributed on both sides of the average effect size, and most of the effect sizes were distributed in the upper middle, with fewer effect sizes at the bottom. This shows that the possibility of publication bias in the study is quite small. Secondly, the Egger’s regression test showed a *p*-value of 0.98, which is far greater than 0.05. This indicates that there is no publication bias in this study (Egger et al., 1997).

### 3.3 Moderating variables analysis

The overall analysis results show there to be significant heterogeneity in the influence parental involvement has on math performance. To gain a deeper understanding of the factors that affect the relationship between parental involvement and math performance, this study conducted moderating variable analysis on father or mother, involvement type, grade level, geographical region, math performance evaluation type, evaluation content, and publication year to determine whether parental involvement affects student mathematical performance differently in different contexts. The results of the moderating variables analysis can be seen in Table 4.

**Table 4:** Moderating variables analysis.

Moderating variables	*Q*	k	*r*	95%CI	*p*
Participant	6.557				<0.05
All		37	0.135	[0.096, 0.174]	
Father		2	0.064	[0.017, 0.111]	
Mother		3	−0.090	[−0.394, 0.235]	
Involvement type	193.961				<0.05
General involvement		11	0.166	[0.096, 0.233]	
Homework assistance		7	−0.143	[−0.220, −0.065]	
Involvement at home		7	0.122	[−0.021, 0.259]	
Involvement at school		7	0.087	[0.021, 0.151]	
Parental expectation		7	0.335	[0.315, 0.354]	
Parental support		3	0.213	[0.038, 0.375]	
Grade level	18.800				<0.05
Preschool		3	0.116	[0.018, 0.221]	
Primary school		24	0.110	[0.048, 0.172]	
Middle school		11	0.135	[0.054, 0.214]	
High school		3	0.187	[0.006, 0.356]	
University		1	−0.146	[−0.258, −0.030]	
Geographical region	9.716				<0.05
Asia		7	0.130	[−0.062, 0.312]	
USA		22	0.109	[0.036, 0.180]	
Europe		5	−0.049	[−0.235, 0.140]	
Other		8	0.242	[0.154, 0.326]	
Evaluation type	3.317				>0.05
Standardized test		32	0.089	[0.048, 0.130]	
Custom test		10	0.228	[0.084, 0.364]	
Evaluation content	46.851				<0.05
General knowledge		28	0.090	[0.046, 0.134]	
Concepts and number		4	0.149	[0.047, 0.248]	
Calculations		4	−0.005	[−0.303, 0.295]	
Problem-solving		1	0.383	[0.217, 0.528]	
Algebraic reasoning		3	0.265	[0.077, 0.435]	
Spatial sense		1	0.234	[0.055, 0.399]	
Number sense		1	0.431	[0.335, 0.518]	

#### 3.3.1 Participant

The analysis results show that there were differences in the effect size of participant on math performance, which indicates a moderating effect (*Q* = 6.557, *p <* 0.05). More specifically, the effect size of parental joint involvement in the study was found to be the largest (*r* = 0.135), followed by father involvement (*r* = 0.064), while the effect size of mother involvement showed a negative correlation (*r* = −0.090).

#### 3.3.2 Involvement type

The analysis results show that involvement type had a moderating effect on the relationship between parental involvement and the math performance of students (*Q* = 193.961, *p* < 0.05). The effect sizes from high to low are parental expectation (*r* = 0.335), parental support (*r* = 0.213), general involvement (*r* = 0.166), involvement at home (*r* = 0.122), and involvement at school (*r* = 0.087), while homework assistance had the worst effect and was found to have a negative correlation with math performance (*r* = −0.143).

#### 3.3.3 Grade level

The analysis results show that grade level had a moderating effect on the relationship between parental involvement and math performance (*Q* = 18.800, *p* < 0.05). More specifically, high school had the highest effect size (*r* = 0.187), followed by middle school (*r* = 0.135). The effect sizes for primary school (*r* = 0.110) and preschool (*r* = 0.116) were similar, while university effect size was the worst (*r* = −0.146).

#### 3.3.4 Geographical region

This study divided geographical regions into four categories: United States, Europe, Asia, and other countries. The analysis results show there to be differences in the effect sizes (*Q* = 9.716, *p* < 0.05), and geographical location was also a moderating factor that affected the relationship between parental involvement and the math performance of students. The Asian samples had the largest effect size (*r* = 0.130), followed by US samples (*r* = 0.109), while the European samples had the worst effect size, showing a negative correlation (*r* = −0.049). However, considering the small sample sizes in Asia and Europe, we supposed that this moderating effect was not representative.

#### 3.3.5 Evaluation type

The analysis results show that evaluation type has no effect on the relationship between parental involvement and the math performance of students (*Q* = 3.317, *p* > 0.05). However, when using custom test to evaluate student math performance (*r* = 0.228), the correlation was found to be stronger than with standardized test (*r* = 0.089).

#### 3.3.6 Evaluation content

The analysis results show that different evaluation contents affected the relationship between parental involvement and the math performance of students (*Q* = 46.851, *p* < 0.05). In comparison to basic mathematical knowledge such as concepts and numbers (*r* = 0.149), the correlation between parental involvement and advanced mathematical knowledge, such as problem-solving (*r* = 0.383) and algebraic reasoning (*r* = 0.265) was stronger.

#### 3.3.7 Publication year

The results of the meta-regression analysis can be seen in Table 5 and they show that year of publication had no moderating effect on the relationship between parental involvement and the math performance of students.

**Table 5:** Meta-regression analysis.

	Variable	Coefficient	SE	95%CI	*p*-value
PI	Publication year	0.0016	0.0075	[−0.0131, 0.0163]	0.8277
		−3.1791	15.1367	[−32.8464, 26.4883]	0.8336

PI, Parental involvement.

## 4 Discussion

This study analyzed 25 articles with 42 independent effect sizes, and the results showed parental involvement to have a significantly positive influence on the math performance of students. The analysis of moderating variables found that participant, involvement type, grade level, geographical region, and evaluation content can moderate this influence.

### 4.1 Parental involvement and the math performance of students

Meta-analyses on the relationship between parental involvement and academic performance in recent years have found parental involvement to have a positive influence on the academic performance of students (Castro et al., 2015; Erdem and Kaya, 2020; Ates, 2021; Wilder, 2023). Henderson and Mapp (2002) noted that when schools, families, and communities jointly support learning, children are more likely to enjoy learning more and do better. When parents are involved in the education of their children, students will feel that their parents provide them with a sense of security and connection in their studies, which helps enhance their learning motivation (Gonzalez-DeHass et al., 2005). At the same time, parental involvement plays a vital role in the emotional and cognitive development of adolescents (Hampden-Thompson and Galindo, 2017; Berkowitz et al., 2021).

Math is essential for national development and many countries have implemented policies to promote math teaching and learning as a means of improving the quality of math education (Muraina and Ajayi, 2011). However, many students continue to face many difficulties in math learning, which leads them to lose confidence and causes anxiety (Mamolo, 2022). In comparison to math, literacy and language skills receive more attention from parents as they often think that their mathematical abilities are weak and that they cannot provide assistance to their children in mathematical learning (Berkowitz et al., 2021). Some parents even hope that teachers and schools take sole responsibility for the mathematical learning of their children (Cannon and Ginsburg, 2008). This study conducted a meta-analysis on the influence parental involvement has on math performance, the results showing that parental involvement has a significantly positive influence on it, which indicates that parental involvement is necessary for mathematical learning. Panaoura (2017) noted that parental involvement can help students develop the confidence and perseverance that are needed for mathematical learning. Parental involvement is essential for helping adolescents develop their self-efficacy (Schunk and Miller, 2002). Students with low self-efficacy may avoid math that they perceive to be difficult, while students with high self-efficacy may persist in math learning while obtaining satisfaction, which will encourage them to perform better in math (Margolis and McCabe, 2004; Schunk and Zimmerman, 2006). Therefore, parents should be advocated and encouraged to become involved in the math learning of their children.

### 4.2 Moderating effects

Seven moderating variables were chosen in this study. The analysis results showed there to be differences in participant, involve type, grade level, geographical regions, and evaluation content.

#### 4.2.1 Participant

The analysis result showed the joint involvement of father and mother to have a stronger influence on the math performance of students than father involvement, while mother involvement was found to have a negative influence. As is widely known, a good family environment cannot be separated from the mutual cooperation between father and mother (Krauss et al., 2020). Most people considered that the responsibility for managing the academic performance of children lies with the mother, while the role of the father is often neglected (Rodriguez and Tamis-LeMonda, 2011). Due to the different ways in which fathers and mothers are involved in the education of their children, the effect of only father or mother involvement is not ideal. Therefore, the joint involvement of father and mother in child education should be promoted.

#### 4.2.2 Involvement type

The analysis results showed parental expectation and parental support to have a stronger correlation with the math performance of students than other involvement types. Parental expectation and support may influence the math performance of students in several ways. Firstly, parental expectation and support can stimulate motivation in children to learn math. Secondly, positive expectations and support from parents in mathematical learning can enhance the self-efficacy and learning abilities of children. Finally, parents with high expectations for their children regarding math performance will help them in various aspects, such as actively communicating with their children emotionally and strengthening connections with schools and teachers, which will help students achieve better mathematical learning performance (Kung and Lee, 2016; Rodríguez Martínez et al., 2017; Tan et al., 2020). Therefore, when parents are involved in mathematical learning, they should give their children more positive expectations and support.

The analysis results show that homework assistance had a negative influence on the math performance of students. Regarding the relationship between homework assistance and student academic performance, current research results are inconsistent. The reason for this contradiction may be related to the way parents provide assistance for the homework of their children (Pomerantz and Eaton, 2001; Patall et al., 2008; Hill and Tyson, 2009; Silinskas et al., 2013). When parents impose mandatory assistance and supervision on this, it may weaken the abilities and confidence of their children in mathematical learning, which could lead to them becoming more reliant on their parents (Pomerantz and Eaton, 2001; Viljaranta et al., 2018). If parents give their children autonomy in homework assistance, which would allow them to think independently and solve problems, their mathematical learning ability and confidence would improve as a result, enabling them to achieve better mathematical performance (Silinskas et al., 2015). Therefore, parents should pay attention to the way they are involved in math homework assistance.

#### 4.2.3 Grade level

The analysis results show parental involvement to have the greatest positive influence on the math performance of students in high school, followed by middle school, while parental involvement was found to have the weakest positive influence in primary school and preschool. The reason for these results may be that the mathematical content learned by students in preschool and primary schools is relatively basic, and parents do not need to master professional mathematical knowledge and skills (Szczygiel, 2020). Therefore, parents involve in their children’s mathematical learning is mostly through homework assistance and intervention, which weakens students’ autonomy in learning and has a negative influence on their math performance. In middle and high school, the difficulty of students’ mathematical knowledge increases, so parents involve in their children’s mathematical learning is more about giving expectations and encouragement. This positive involvement enhances emotional communication between parents and children, cultivates students’ autonomy in learning, and has a positive influence on their math performance.

However, the analysis results show there to be a negative correlation between parental involvement and the math performance of students at university. This may be because university students have already passed their teenage years and they hope their parents can give them more autonomy in making choices (Arshad et al., 2016). In addition, most of the parents involved in the study have received higher education and have higher expectations for their children’s academic performance (Abu Bakar et al., 2021). Excessive demands from parents can bring pressure to students, which have a negative influence on their math performance.

#### 4.2.4 Evaluation content

The analysis results show that the evaluation content will affect the relationship between parental involvement and math performance. With basic math knowledge, including concepts and calculations, students find it relatively easy to learn. With advanced math knowledge, such as problem-solving and algebraic reasoning, students have certain difficulties learning, so they need help from their parents (Hewson, 2011; Santana et al., 2019). Therefore, parental involvement has a stronger positive influence on student performance in advanced math knowledge.

Geographical region had a moderating effect on the relationship between parental involvement and students’ math performance. However, we considered that this moderating effect may be caused by uneven sample size distribution. In future research, we will retest the moderating effect of geographical region based on richer data.

The analysis results show evaluation type and publication year to have no moderating effect, but standardized test can avoid the influence of subjective factors and is therefore superior to custom test. At the same time, more policies and measures promoting parental involvement in the mathematical learning of students are required.

## 5 Implications

Through meta-analysis results, it was found that parental involvement can have a positive influence on students’ math performance. At the same time, there were also some problems in parental involvement in students’ mathematical learning that need to be solved in the future. According to SDT, positive parental involvement plays an important role in cultivating students’ learning autonomy, and parental involvement can enhance interaction and emotional communication with children (Ryan and Deci, 2000). Therefore, the quality of parental involvement should be given attention.

Firstly, the father and mother need to work together to create a good family learning environment. Secondly, parents should pay attention to the way they involve in their children’s mathematical learning, and provide students with more guidance and encouragement, cultivating autonomy of students in mathematical learning. Finally, parents should actively communicate with schools and teachers to gain a comprehensive understanding of their children’s math performance, in order to adjust their strategies for involving in students’ mathematical learning.

In addition, schools and teachers play an important role in improving the quality of parental involvement. Schools can design and hold family education workshops to provide guidance and training for parents in family education, enhance the emotional communication ability between parents and children, and enable parents to better involve in their children’s mathematical learning. Math teachers can provide supportive counseling or guidance strategies to help parents involve more effectively in their children’s math homework assistance.

## 6 Limitations and prospects

Although this study comprehensively analyzed the influence parental involvement has on the math performance of students, there are some limitations. Firstly, this study only searched for English literature and did not consider literature in any other languages, so there is a shortage in the amount of literature that is included in the study. Secondly, there is a lack of unified standards for classifying and evaluating parental involvement. This study categorizes based on the reported content in the included literature, so there are shortcomings in terms of classification standards. Finally, regarding moderating variable analysis, some of the moderating variables examined that are in this study have significant sample size differences, which will affect the results of moderating variable analysis.

Future research could expand the scope of literature inclusion to include literature in other languages, which would expand the sample size and make the meta-analysis results more comprehensive. Secondly, it should further unify the classification criteria for parental involvement and develop standardized evaluation scales. Finally, the sample population of this study is mainly primary and secondary schools and physically healthy students, so research could also be conducted on university students and students with physical disabilities.

## 7 Conclusion

This study conducted a meta-analysis of 25 studies and found parental involvement to have a significantly positive influence on the math performance of students. At the same time, this influence was moderated by participant, involvement type, grade level, geographical region, and evaluation content, but had no relationship with evaluation type and publication year.

## Data Availability

The original contributions presented in the study are included in the article/supplementary material, further inquiries can be directed to the corresponding author.
